# Derivation of a Risk Score for High Caries Risk in 3- to 5-year-old Children in Sichuan Province

**DOI:** 10.3290/j.ohpd.b1452865

**Published:** 2021-06-01

**Authors:** Lei Lei, Bo Yuan, Hong Chen, Ying-Ming Yang, Tao Hu

**Affiliations:** a Associate Professor, State Key Laboratory of Oral Diseases and National Clinical Research Center for Oral Disease and Department of Preventive Dentistry, West China Hospital of Stomatology, Sichuan University, Chengdu, China. Collected and interpreted the data, drafted the article and revised it critically for important intellectual content, read and approved the final manuscript.; b Dentist, Department of Stomatology, Longhua People′s Hospital Affiliated to Southern Medical University, Shenzhen, China. Collected and interpreted the data, read, revised and approved the final manuscript.; c Lecturer, State Key Laboratory of Oral Diseases and National Clinical Research Center for Oral Disease and Department of Preventive Dentistry, West China Hospital of Stomatology, Sichuan University, Chengdu, China. Collected data, read, revised and approved the final manuscript.; d Associate Professor, State Key Laboratory of Oral Diseases and National Clinical Research Center for Oral Disease and Department of Preventive Dentistry, West China Hospital of Stomatology, Sichuan University, Chengdu, China. Involved in study conception and design, acquisition of data, analysis and interpretation of data; drafted the manuscript, read and approved the final manuscript.; e Professor, State Key Laboratory of Oral Diseases and National Clinical Research Center for Oral Disease and Department of Preventive Dentistry, West China Hospital of Stomatology, Sichuan University, Chengdu, China. Read, revised and approved the final manuscript.

**Keywords:** children, dental caries, epidemiology, high risk, risk score model

## Abstract

**Purpose:**

To explore potential caries risk indicators in 3- to 5-year-old children, and develop a simple risk-score model to screen the children at high risk of caries with decayed, filled, and missing teeth (dmft) > 2.

**Materials and Methods:**

A cross-sectional study involving 2746 children 3 to 5 years of age was conducted in Sichuan province. Children were examined for dmft index, and sociodemographic and behavioural factors were acquried through a questionnaire completed by their caregivers. A prediction model was developed by backward multivariate logistic regression, and its overfitting degree was examined with 5-fold cross-validation. A simple risk-score model was derived to screen the children with dmft > 2 at high risk of caries with the β regression coefficient obtained from the multivariate regression model.

**Results:**

A child’s oral health status was identified as the highest risk indicator with a β regression coefficient of 1.093. The mean area under curve (AUC) from the 5-fold cross-validation was 0.7408 (95% CI: 72.21%, 75.95%), with a bias of only ca 1%. This result allowed us to eliminate substantial overfitting of the prediction model. The AUC of the risk scoring system was 0.7455 (95% CI: 72.70%, 76.40%), which indicated good screenability.

**Conclusions:**

This risk score model has the advantages of simplicity, low cost and relatively high accuracy, and is suitable for use in developing countries, especially for primary screening for high risk of caries. It shows that certain child behaviours and parental attitude play an important role in dental caries among preschool children.

Early childhood caries (ECC) is the most prevalent chronic infectious childhood disease and is a major public health problem.^19^ ECC is defined by the American Academy of Pediatric Dentistry (AAPD) as the presence of one or more decayed (non-cavitated or cavitated lesions), missing (due to caries), or filled tooth surfaces in any primary tooth in a child under six years old.^2^ Although ECC incidence has declined in some developed countries, it remains a serious problem in developing countries.^4^ ECC is also becoming a diagnostic challenge, with changing diet and nutrition.^6^ A study has shown a high prevalence in Asia (36%–85%), Africa (38%–45%) and the Middle East (22%–61%).^4^ ECC prevalence was 65.5% and 66.1% in mainland China among 1- to 6-year-olds and 5-year-olds, respectively.^37^ The World Health Organization’s (WHO) target is that half of 6-year-old children should be caries-free.^9^ To meet this criterion, many developing countries need to make a great effort. Several studies have reported that ECC can influence children’s quality of life, including oral health, general health, growth, and even the quality of life of children’s families and communities.^4,21^ All of those studies have emphasised that poor oral health is an additional burden for children’s health and underlined the importance of caries prevention in children.

Interestingly, there is a skewed caries distribution in many developed countries, with 25% of children bearing 75%–80% of affected surfaces.^11,20^ Therefore, the National Institutes of Health (NIH) pointed out that caries prevention should target high-risk individuals.^18^ In this study, a dmft index of two or more decayed, missing or filled primary teeth for 3- to 5-year-old children used to define high risk of dental caries according Gao’s criteria.^7^ Early, precise and low-cost selection of high-risk preschoolers through caries risk assessment for prevention and intervention is paramount for cost-effective caries control. Several conceptual models have been proposed by professional organisations, such as the Caries-risk Assessment Tool proposed by the International Caries Detection and Assessment System (ICDAS),^1^ and the Caries Management by Risk Assessment programme advocated by the California Dental Association.^5^ According to the data from China’s National Health and Family Planing Commission, approximately 220,000 dental practitioners and assistants were available in 2018,^36^ but there are 1.4 billion people and more than 20 million children aged 3–5 years in China.^15^ This means that less than one dental practitioner and assistant is available for every 1000 people. Consequently, it is very difficult for dental practitioners and assistants to adequately screen such a large number of children. The aim of this study was to explore potential caries risk indicators, develop a simple risk-score model to screen the children with dmft > 2 for high risk of caries, and in the future provide more public health focus and dental resources to these in children with a higher risk.

## Materials and Methods

### Sampling

Data were collected through oral examination and a questionniare with children and their caregivers at the kindergartens in Sichuan Province. Sichuan Province, located in southwest China, is inhabited by multiple ethnic groups, and has a population of 81.4 million people.^35^ The economic aggregate of Sichuan ranks first in western China and sixth in China overall. Three oral health surveys were conducted in Sichuan in 1983, 1995, and 2005. A complex, multistage, cluster sampling design^30^ was performed based on the Fourth National Oral Health Survey and a previous study.^34^ Six areas (Guang’an District, Chuan’shan District, Jin’niu District, Da County, Yi’bin County, and Pi County) were selected for this study. Then, three kindergartens were ramdomly selected by probabilities proportional to size in each area.^13^ Finally, children in the selected kindergartens were chosen using a quota sampling method. According to the following equation, the required sample size was 2472.


$$n=\text{ deff }\frac{\mu^{2}(1-p)}{\varepsilon^{2}p(1-\text{ nonresponse })}$$

where n is the sample size, deff is the design effect (2.5), p is the prevalence of dental caries (66.0%) in children aged 3–5 years from the Third National Oral Health Survey,^32^ μ is the level of confidence, and ε is the margin of error. The predicted non-response rate was 20% in 3–5 years old children.^34^ Finally, 2746 children aged 3–5 years were selected in this study, which was slightly greater than the required sample size (2472). Approval was obtained from the Stomatological Ethics Committee of the Chinese Stomatological Association and the Ethics Committee of West China Hospital of Stomatology, Sichuan University (Approval No. 2014-003), and all caregivers of the children were required to sign an informed consent form.

### Clinical Assessment

The caregivers of all the children enrolled in this study signed the informed consent form. The children received a clinical examination according to the basic methods and criteria issued by the WHO Oral Health Survey. The content of the clinical assessment included the numbers of decayed, missing and filled teeth.^34^ Four trained and accredited dentists performed the examination. The mean Kappa values for the inter-examiner reproducibility was >0.85 for the caries examination.^34^

### Questionnaire

The questionnaire requested the following information: dietary habits, oral hygiene practices, dental attendance, oral health status and caregivers’ oral health knowledge and attitude. To ensure accuracy and reliability, every question in the questionnaire was filled out by trained investigators during a one-to-one interview with the children’s caregivers.

### Statistical Analysis

First, we conducted a univariate logistic regression to screen for the possible risk indicators potentially associated with the outcome variable; and the entry criterion was p < 0.1. The value was used with the aim of minimising residual confounding due to the risk of omitting relevant variables.^23^ Second, a prediction model was developed by a backward multivariate logistic regression. The variables mentioned above were entered into the regression model if p was < 0.05 and removed if p was > 0.1. We calculated the tolerance and variance inflation factor of each covariate to test the collinearity between the covariates of the multivariable model. The Hosmer-Lemeshow goodness-of-fit test was performed to assess the calibration of the regression model. The area under the receiver operating characteristic (ROC) curve (AUC) was calculated to assess the discrimination of the final model. A k-fold cross-validation (k = 5) was conducted to examine the degree of overfitting of the prediction model. The diagnostic performance of the prediction model was assessed by comparing the mean AUC of the ROC from 5-fold cross-validation with that of the observations used to create the model. The β regression coefficient from the prediction model was used to derive a practical scoring system, as shown in previous studies.^28^ We assigned weighted points to the predictors identified with regression analysis proportional to the β regression coefficient values. A risk score was then calculated for each child. The AUC was calculated to validate the predicted performance of the risk-score system. All the analyses were performed using SPSS v 20.0 (SPSS, IBM; Armonk, NY, USA) and R 3.3.1. A two-sided p-value < 0.05 was considered statistically significant.

## Results

### Demographics

A total of 3000 children aged 3-5 years were selected for this study; the non-response rate was 8.45%. Thus, a final number of 2746 children participated in our study.^22,34^ Among the children, 1362 (49.6%) were girls and 1384 (50.4%) were boys. The prevalence of caries was 63.47% (1743) and the mean dmft was 3.28.^22,34^ Among all the participants, 1132 children had a dmft > 2. The results of univariate analysis to select potential indicators associated with the outcome variables (dmft > 2) are summarized in Table 1. Ultimately, ten variables were selected for the regression model with p < 0.1.

**Table 1:** Potential risk indicators selected by univariate analysis (n = 2746)

Variables	Dmft > 2 (n%)	dmft ≤ 2 (n%)	OR 95% CI	p-value
**Age (years)**				
3	216 (7.87%)	592 (21.56%)	1	
4	397 (14.46%)	524 (19.08%)	2.08 (1.70, 2.54)	< 0.0001
5	519 (18.90%)	498 (18.14%)	2.86 (2.34, 3.48)	< 0.0001
**Household type**				
Non-agricultural family	295 (10.74%)	569 (20.72%)	1	
Agricultural family	837 (30.48%)	1045 (38.06%)	1.55 (1.31,1.83)	< 0.0001
**Relatives**				
Grandparents	501 (18.24%)	772 (28.11%)	1	
Parents	631 (22.98%)	842 (30.66%)	1.16 (0.99, 1.35)	0.065
**Sugar-containing soft drink/soda consumption**				
≤ 1/week	836 (30.44%)	1301 (47.38%)	1	
> 1/week	296 (10.78%)	313 (11.40%)	1.47 (1.23, 1.76)	< 0.0001
**Dessert or sugar-containing drink consumption before sleep**				
Never	510 (18.57%)	823 (29.97%)	1	
Occasionally	454 (16.53%)	572 (20.83%)	1.28 (1.09, 1.51)	0.003
Often	168 (6.12%)	219 (7.98%)	1.24 (0.98, 1.56)	0.068
**Toothache in previous year**				
No or unclear	744 (27.09%)	1460 (53.17%)	1	
Yes	388 (14.13%)	154 (5.61%)	4.94 (4.02,6.08)	< 0.0001
**Dental visit history**				
No	870 (31.68%)	1455 (52.99%)	1	
Yes	262 (9.54%)	159 (5.79%)	2.76 (2.22,3.41)	< 0.0001
**Last dental visit**				
> 12 months ago or never	937 (34.12%)	1502 (54.70%)	1	
6–12 months ago	76 (2.77%)	46 (1.68%)	2.65 (1.82,3.85)	< 0.0001
< 6 months ago	119 (4.33%)	66 (2.40%)	2.89 (2.12,3.95)	< 0.0001
**Child’s oral health status assessment**				
Very good or good	319 (11.61%)	970 (35.32%)	1	
Fair, poor or very poor	813 (29.61%)	644 (23.45%)	3.84 (3.26, 4.52)	< 0.0001
**Oral health knowledge and attitude score**				
High	463 (16.86%)	733 (26.69%)	1	
Medium	590 (21.49%)	806 (29.35%)	1.16 (0.99, 1.36)	0.067
Low	79 (2.88%)	75 (2.73%)	1.67 (1.19, 2.34)	0.003

### Multivariate Logistic Regression

The regression model was used to identify 7 significant (p< 0.05) variables as predictors (Table 2): age, household type, sugar-containing soft drink/soda consumption, toothache in previous year, dental visit history, child’s oral health status assessment (by the parent/caregiver), caregivers’ oral health knowledge and attitude score. Among them, the variable child’s oral health status assessment was identified as the highest risk indicator, with a β regression coefficient of 1.093.

**Table 2:** Logistic regression analysis for the variables related to dmft > 2 and scoring system

Variables	β regression coefficient	OR	95%CI	p-value	Score*
**Age (years)**					
3		1			0
4	0.632	1.88	1.51, 2.34	< 0.0001	6
5	0.863	2.37	1.91, 2.94	< 0.0001	9
**Household type**					
Non-agricultural family		1			0
Agricultural family	0.428	1.53	1.27, 1.85	< 0.0001	4
**Sugar-containing soft drink/soda consumption**					
≤1/week		1			0
>1/week	0.303	1.35	1.11, 1.66	0.003	3
**Toothache in previous year**					
No or unclear		1			0
Yes	1.064	2.90	2.30, 3.66	< 0.0001	11
**Dental visit history**					
No		1			0
Yes	0.379	1.46	1.13, 1.88	0.004	4
**Child’s oral health status assessment**					
Very good or good		1			0
Fair, poor or very poor	1.093	2.98	2.50, 3.55	< 0.0001	11
**Oral health knowledge and attitude score**					
High		1			0
Medium	0.099	1.10	0.93, 1.32	0.27	1
Low	0.494	1.64	1.13, 2.38	0.009	5

A reference risk indicator profile was selected by choosing a reference category for each risk indicator. The reference category was the category corresponding to 0 points in the scoring system. Risk indicators for poor health were assessed by a positive score, so that a higher point total conveys more risk. *Score assignment to risk indicators was based on a linear transformation of the corresponding β regression coefficient. The coefficient of subclassification of each variable was divided evenly by 0.099 (the lowest β value), except the reference category, multiplied by a constant (1), and rounded to the nearest integer.

The tolerance of variables in the final multivariable model ranged from 0.79 to 0.98; the mean variance inflation factor was 1.10 (range: 1.01–1.27). The Hosmer-Lemeshow goodness-of-fit test statistic was 0.653, indicating good model calibration.

### Cross-validation

A stratified k-fold (k = 5) cross-validation was performed by dividing the data into five parts. The mean AUC of the 5 k-fold cross-validation was 0.7408 (95% CI: 72.21%, 75.95%). The AUC for the prediction model built by all data was 0.7458 (95% CI: 72.73%, 76.43%), indicating a bias of about 1%. Hence, this result allowed us to eliminate substantial overfitting of the prediction model. In order to test the stability and reproducibility of the model with all the indicators, we investigated the five models created during cross-validation. The risk predictors such as age, household type, sugar-containing soft drink/soda consumption, toothache in previous year, dental visit history, and child’s oral health status assessment occurred in all the models and the indicator ‘caregiver oral health knowledge and attitude’ score occurred in three of the five models.

### Risk Scoring System

A number of points were assigned to each category of the seven predictor variables proportional to the β regression coefficient to calculate the risk score (Table 2). When the β-coefficient of different variables (e.g. 3-year-old children, children of non-agricultural family, etc) in the multivariable model was used as a reference, although the scoring system was different, the discrimination of the final model was similar (data not shown). Table 3 shows the total point (range: 0-50) and corresponding estimated risk.

**Table 3:** Total points and the corresponding risk estimation

Total points	Risk estimation	Total points	Risk estimation
0	0.0972	30	0.6552
5	0.1379	35	0.7571
10	0.2078	40	0.8364
15	0.3009	45	0.8935
20	0.4139	50	0.9383
25	0.5367		

Risk estimation = 1/(1 + EXP [- 2.229 + 0.099 (total points)]). In our study, the lowest score was 0 and the highest was 47. In this table, only some numerical scores and their corresponding estimate of risk were given.

**Fig 1:**
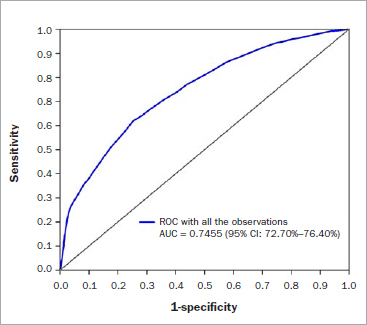
ROC curve developed using the weighted score. The AUC of the risk scoring system was 0.7455 (95% CI: 72.70%, 76.40%), indicating good screenability.

A score was calculated for each child by summing the points that corresponded to the risk indicators. We applied this scoring system to all children participating in this study (see Table 2 for application). An ROC curve was developed by using the weighted score (Fig 1). The AUC was 0.7455 (95% CI: 72.70%, 76.40%), showing good screenability of the risk-score system. The screen performance of the weighted score for determining dmft > 2 is shown in Table 4. The threshold score was 20.5, i.e. if the score was > 20.5, the child dmft might be > 2.

**Table 4:** Summary findings of the risk scoring system applied to all children

Performance measures	Estimation	Lower confidence limit	Upper confidence limit
Sensitivity	0.6201	0.5911	0.6485
Specificity	0.7460	0.7240	0.7671
PPV	0.6313	0.6022	0.6597
NPV	0.7368	0.7148	0.7581
LR+	2.4412	2.2195	2.6851
LR-	0.5092	0.4702	0.5515
Youden index	0.3661	0.3308	0.4015
Accuracy	0.6941	0.6765	0.7113

The threshold score was 20.50. PPV: positive predictive value; NPV: negative predictive value; LR+: positive likelihood ratio; LR-: negative likelihood ratio.

## Discussion

In this study, the variables obtained from clinical examination and a questionnaire survey of the enrolled children were systematically analysed and the risk indicators with dmft > 2 were determined. According to our results, age, household type, sugar-containing soft drink/soda consumption, toothache in previous year, dental visit history, child’s oral health status assessment, caregivers’ oral health knowledge and attitude scores resulted in independent risk indicators with dmft > 2, and the variable child’s oral health status assessment was identified as the highest risk indicator, with a β regression coefficient of 1.093.

The prediction model showed good discrimination and calibration, and included the accepted variables for caries in children. We further converted the regression coefficient to a point-based scoring system to simplify screening for a high risk of caries (dmft > 2) in 3- to 5-year-old children; this method can be applied in developing countries for oral health surveys including primary screening. The point-based score derived by combining points for each of the indicators can be used for determine high risk of caries. As shown in Table 2, the researchers were able to obtain relevant data through questionnaires and then calculate the scores for each child based on the score of the sub-category score of each variable. As shown in Table 3, we evaluated the caries risk for each child. The advantages of our risk-scoring system are: (a) all the variables can be easily obtained by a simple questionnaire which contains a simple calculation at its end; (b) a minimum amount of clinical information was required; and (c) estimation of the specific high caries risk in a child by using a nomogram reference was possible.

In the present study, age was an important risk indicator (β regression coefficients 0.632 and 0.863 for 4 and 5 years, respectively), which has a high proportion of weight in the risk scoring system. This result resembled that found by Prakash et al.^21^ The potential reason was the effect of a combination of unhealthy dietary habits and bad oral hygiene, over time leading to tooth decay and caries experience, which increased with age. Previous studies showed that the consumption of sugar-containing foods or beverages,^8^ lower socioeconomic status,^12^ toothache experience,^26,31^ caregiver’s assessment that the child’s oral health is poor,^25,26^ and little knowledge of and negative attitude toward oral health^14^ increased the likelihood of caries. The present study did not include the ‘income’ variable, due to 361 answered questionnaires lacking income data. Generally, children who have visited a dentist should have less caries experience compared to those who have not. In this study, however, the opposite was true. We suspect that people in low-income countries seek medical or dental treatment when they experience discomfort or cannot tolerate the pain, which makes dental visit experience a risk indicator rather than a protective indicator. Other studies^24,26^ have shown similar results. The univariate logistic regression analyses showed the p-value of the variables regarding brushing habits and caregivers’ educational level to be > 0.1, indicating that these factors might not be risk indicators of the outcome variable. These findings did not agree with those of other authors.^3,17^ Incorrect brushing methods might explain this difference between the current results and those of previous studies. Interestingly, caregivers’ education level was not a risk indicator in our study. We postulate that the reason lies in predominantly poor health awareness, particularly regarding oral health, in many developing countries, including China.^33^ Nevertheless, another study found caregivers’ oral health knowledge attitude to be an important risk indicator.^14^ Logically, if the caregivers judged the child’s oral health to be poor or they realised that the child experienced toothache, a higher risk of caries might be present. It was striking that the children’s caregivers were aware of children’s oral health status, but filled teeth accounted for less than 5% of the total number of dmf teeth in our study. It is possible that the caregivers lacked access to dental services or just guessed because they did not understand the questions. In order to solve this problem and obtain obtain more accurate questionnaire results in the future and in other developing countries, it is important to enhance public oral-health education, and the investigator needs to explain the questionaire in more detail to the respondents.

To be effective, a risk-assessment programme should be simple and possess both high sensitivity and specificity.^27^ However, with the trade-off between simplicity and accuracy, it may be impractical to achieve both simultaneously. Our study presents a simple, low-cost primary screening model with relatively high accuracy. The common caries risk assessments used in economically advanced country are CAMBRA (caries management by risk assessment), CAT (caries risk assessment tool) and Cariogram (caries risk assessment programme). However, these methods have some disadvantages. CAT and CAMBRA are verification forms composed of important risk indicators to qualitatively estimate individual risk. However, previous research has shown CAT and CAMBRA to have high sensitivity but low specificity, resulting in overestimating caries risk in children,^1^ certainly causing overtreatment and inefficient use of medical resources. The enormous number of tables and guidelines, e.g. CAMBRA, might in fact increase the difficulty of performing caries risk assessment in children. Although CAT is easier to use, unfortunately, some of the items are unsuited to developing countries. Cariogram simplified the process of assessment, but its accuracy was limited to pre-school children.^10,16,29^ Our risk score model is derived from Sichuan, China, and could be more suitable in developing countries for the primary screening of high caries risk.

### Study Limitations

First, we used a cross-sectional study design. We used categorical variables and a limited number of variables instead of continuous variables to simplify the creation of a risk score. But compared to a longitudinal study and continuous variable acquistion, a cross-sectional study and categorical variable acquisition can save substantial amounts of time and costs. Secondly, some significant variables might not have been included in the model, as variable selection was performed hypothetically. It is not realistic for a model to include all the variables, and we could not increase the sensitivity of a model indefinitely. Finally, the cross-validation might have eliminated model overfitting due to differences between the study sample and the underlying population, but not the overfitting that might arise from differences between patient populations. This is aslo due to sampling and cannot be attributed solely to our model.

## Conclusion

The present study developed a simple risk-score model to screen 3- to 5-year-old children at high risk of caries with dmft> 2 by using the β regression coefficient obtained from a multivariate regression model. This risk-score model has the advantages of simplicity low cost and relatively high accuracy, making it suitable for use in developing countries, especially in primary screening for high risk of caries.

## References

[ref1] Adiningrat A, Kusmaharani HA, Utami S, Ratna Astuti N (2020). Evaluation of International Caries Detection and Assessment System (ICDAS)-related caries severity among caries risk groups in Pendul District: an observational study. J Int Soc Prev Community Dent.

[ref2] American Academy of Pediatric Dentistry (2016). Policy on early childhood caries (ECC): classifications, consequences, and preventive strategies Reference manual. Pediatr Dent.

[ref3] Anil S, Anand PS (2017). Early childhood caries: prevalence, risk factors, and prevention. Front Pediatr.

[ref4] Colak H, Dülgergil CT, Dalli M, Hamidi MM (2013). Early childhood caries update: a review of causes, diagnoses, and treatments. J Nat Sci Biol Med.

[ref5] Featherstone JD, Adair SM, Anderson MH, Berkowitz RJ, Bird WF, Crall JJ (2003). Caries management by risk assessment: consensus statement. J Calif Dent Assoc.

[ref6] Folayan M, Olatubosun S (2018). Early childhood caries –a diagnostic enigma. Eur J Paediatr Dent.

[ref7] Gao XL, Hsu CY, Xu Y, Hwarng HB, Loh T, Koh D (2010). Building caries risk assessment models for children. J Dent Res.

[ref8] Gonçalves EM, Cavalcanti LY, Firmino RT, Ribeiro GL, Granville-Garcia AF, Menezes VA (2015). Dental caries experience among indigenous children and adolescents. J Oral Sci.

[ref9] Hobdell MH, Myburgh NG, Kelman M, Hausen H (2000). Setting global goals for oral health for the year 2010. Int Dent J.

[ref10] Holgerson PL, Twetman S, Stecksèn-Blicks C (2009). Validation of an age-modified caries risk assessment program (Cariogram) in preschool children. Acta Odontol Scand.

[ref11] Kaste LM, Selwitz RH, Oldakowski RJ, Brunelle JA, Winn DM, Brown LJ (1996). Coronal caries in the primary and permanent dentition of children and adolescents 1-17 years of age: United States, 1988–1991. J Dent Res.

[ref12] Kumar S, Tadakamadla J, Zimmer-Gembeck MJ, Kroon J, Lalloo R, Johnson NW (2017). Parenting practices and children’s dental caries experience: a structural equation modelling approach. Community Dent Oral Epidemiol.

[ref13] Lai H, Su CW, Yen AM, Chiu SY, Fann JC, Wu WY (2015). A prediction model for periodontal disease: modelling and validation from a national survey of 4061 Taiwanese adults. J Clin Periodontol.

[ref14] Laksmiastuti SR, Budiardjo SB, Sutadi H (2017). Validated questionnaire of maternal attitude and knowledge for predicting caries risk in children: epidemiological study in North Jakarta, Indonesia. J Int Soc Prev Community Dent.

[ref15] Liu AH, Ye ZC (2020). Population In: National Bureau of Statistics of the People’s Republic of China. China statistic yearbook.

[ref16] Mejàre I, Axelsson S, Dahlén G, Espelid I, Norlund A, Tranæus S (2014). Caries risk assessment. A systematic review. Acta Odontol Scand.

[ref17] Mwakayoka H, Masalu JR, Kikwilu EN (2017). Dental caries and associated factors in children aged 2-4 years old in Mbeya City, Tanzania. J Dent (Shiraz).

[ref18] National Institutes of Health consensus development conference statement (2001). Presented at the Consensus Development Conference on Diagnosis and Management of Dental Caries Throughout Life. J Am Dent Assoc.

[ref19] Ozsin Ozler C, Cocco P, Cakir B (2020). Dental caries and quality of life among preschool children: a hospital-based nested case-control study. Br Dent J.

[ref20] Pitts NB, Chestnutt IG, Evans D, White D, Chadwick B, Steele JG (2006). The dentinal caries experience of children in the United Kingdom, 2003. Br Dent J.

[ref21] Prakash P, Subramaniam P, Durgesh BH, Konde S (2012). Prevalence of early childhood caries and associated risk factors in preschool children of urban Bangalore, India: a cross-sectional study. Eur J Dent.

[ref22] Qin YD, Zhang R, Yuan B, Xu T, Chen H, Yang YM (2019). Structural equation modelling for associated factors with dental caries among 3–5-year-old children: a cross-sectional study. BMC Oral Health.

[ref23] Rijnhart JJM, Twisk JWR, Eekhout I, Heymans MW (2019). Comparison of logistic-regression based methods for simple mediation analysis with a dichotomous outcome variable. BMC Med Res Methodol.

[ref24] Rodrigues LA, Martins AM, Silveira MF, Ferreira RC, Souza JG, da Silva JM (2014). The use of dental services among preschool children: a population-based study. Cien Saude Colet.

[ref25] Scarpelli AC, Paiva SM, Viegas CM, Carvalho AC, Ferreira FM, Pordeus IA (2013). Oral health-related quality of life among Brazilian preschool children. Community Dent Oral Epidemiol.

[ref26] Souza JG, de Barros Lima Martins AM (2016). Dental pain and associated factors in Brazilian preschoolers. Rev Paul Pediatr.

[ref27] Stamm JW, Disney JA, Graves RC, Bohannan HM, Abernathy JR (1988). The University of North Carolina Caries Risk Assessment Study: Rationale and content. J Public Health Dent.

[ref28] Sullivan LM, Massaro JM, D’Agostino RB (2004). Presentation of multivariate data for clinical use: the Framingham study risk score functions. Stat Med.

[ref29] Tellez M, Gomez J, Pretty I, Ellwood R, Ismail AI (2013). Evidence on existing caries risk assessment systems: are they predictive of future caries?. Community Dent Oral Epidemiol.

[ref30] To T, Stanojevic S, Moores G, Gershon AS, Bateman ED, Cruz AA (2012). Global asthma prevalence in adults: findings from the cross-sectional world health survey. BMC Public Health.

[ref31] Wang SS, Zhang H, Si Y, Xu T (2016). Analysis of forecasting indexes for dental caries in 3- to 6-year-old children. Chin J Dent Res.

[ref32] Wang WH, Wang WJ, Wang HY, Kong LZ, Feng XP, Si Y, Qi XQ (2008). Survey results.

[ref33] Wapniarska K, Buła K, Hilt A (2016). Parent’s pro-health awareness concerning oral health of their children in the light of survey research. Przegl Epidemiol.

[ref34] Yin W, Yang YM, Chen H, Li X, Wang Z, Cheng L (2017). Oral health status in Sichuan Province: Findings from the oral health survey of Sichuan, 2015–2016. Int J Oral Sci.

[ref35] Zhang S, Lo EC, Liu J, Chu CH (2015). A review of the dental caries status of ethnic minority children in China. J Immigr Minor Health.

[ref36] Zhang XG, Ma XW, Su G, National Health Commission (2019). Health Personnel.

[ref37] Zhang XL, Yang S, Liao ZY, Yang S, Liao ZY, Xu L (2016). Prevalence and care index of early childhood caries in mainland China: evidence from epidemiological surveys during 1987–2013. Sci Rep.

